# Supplementary material for: Defect passivation in methylammonium/bromine free inverted perovskite solar cells using charge-modulated molecular bonding
Source: Nat Commun. 2024 Jan 29;15:882. doi: 10.1038/s41467-024-45228-9 (PMC10824754; doi:10.1038/s41467-024-45228-9)
Supplement: Supplementary file 1 — Supplementary Information [file 41467_2024_45228_MOESM1_ESM.pdf]

# Supplementary Information

## Defect Passivation in Methylammonium/Bromine Free Inverted Perovskite Solar Cells Using Charge-Modulated Molecular Bonding

Dhruba B. Khadka[1*], Yasuhiro Shirai[1*], Masatoshi Yanagida[1], Hitoshi Ota[2], Andrey Lyalin[3,4*], Tetsuya Taketsugu[4,5], and Kenjiro Miyano[1]

[1] Photovoltaic Materials Group, Center for GREEN Research on Energy and Environmental Materials, National Institute for Materials Science (NIMS), 1-1 Namiki, Tsukuba, Ibaraki 305-0044, Japan

[2]Battery Research Platform, Research Center for Energy and Environmental Materials (GREEN), National Institute for Materials Science (NIMS), Namiki 1-1, Tsukuba 305-0044, Japan

[3]Research Center for Energy and Environmental Materials (GREEN), National Institute for Materials Science, Namiki 1-1, Tsukuba 305-0044, Japan

[4]Institute for Chemical Reaction Design and Discovery (WPI-ICReDD), Hokkaido University, Sapporo 001-0021, Japan

[5]Department of Chemistry, Faculty of Science, Hokkaido University, Sapporo 060-0810, Japan

**Corresponding Author**

*KHADKA.B.Dhruba@nims.go.jp

*SHIRAI.Yasuhiro@nims.go.jp

*lyalin@icredd.hokudai.ac.jp

## Supplemenatry Tables and Supplementary Figures

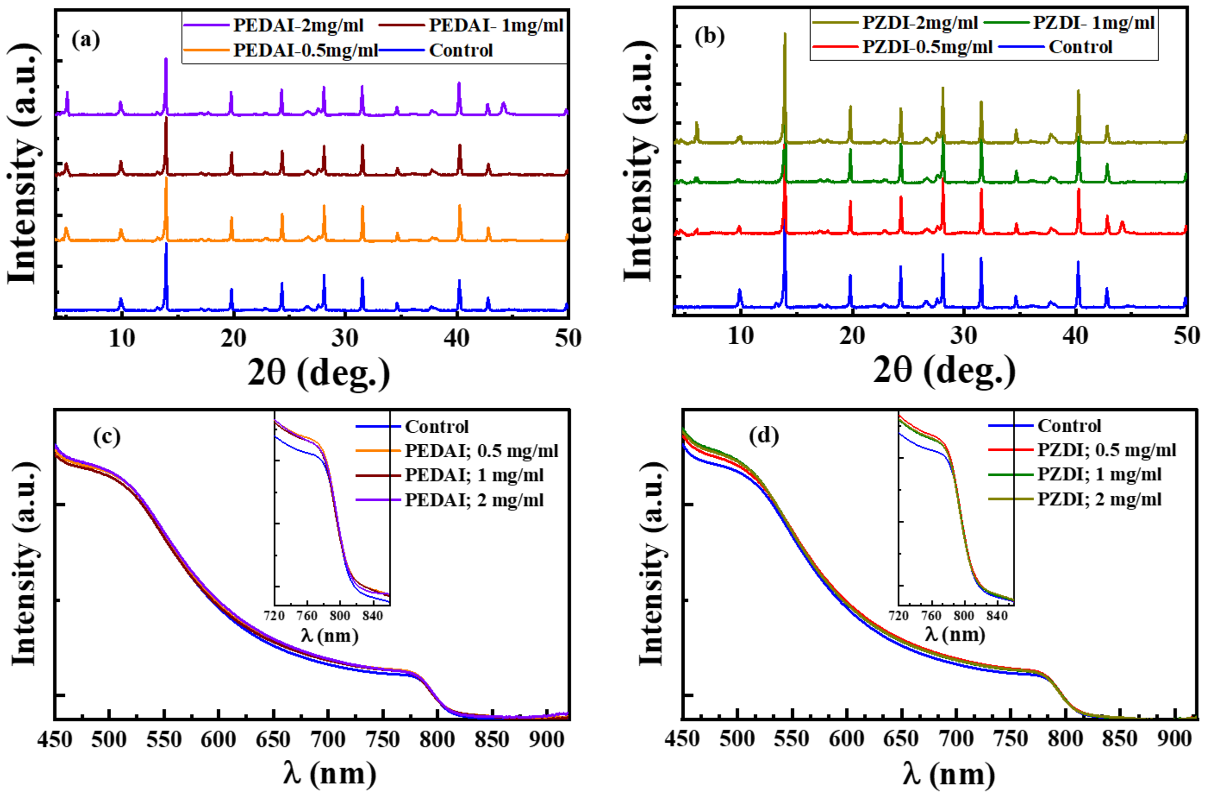


**Fig. S1.** XRD patterns of MA-free ($FA_{0.84}Rb_{0.04}Cs_{0.12}PbI_3$) films without and with DIM treatments: a) PEDAI or b) PZDI solutions in IPA; 0 (control), 0.5, 1, and 2 mg/ml. c, d) Absorption spectra of respective films.

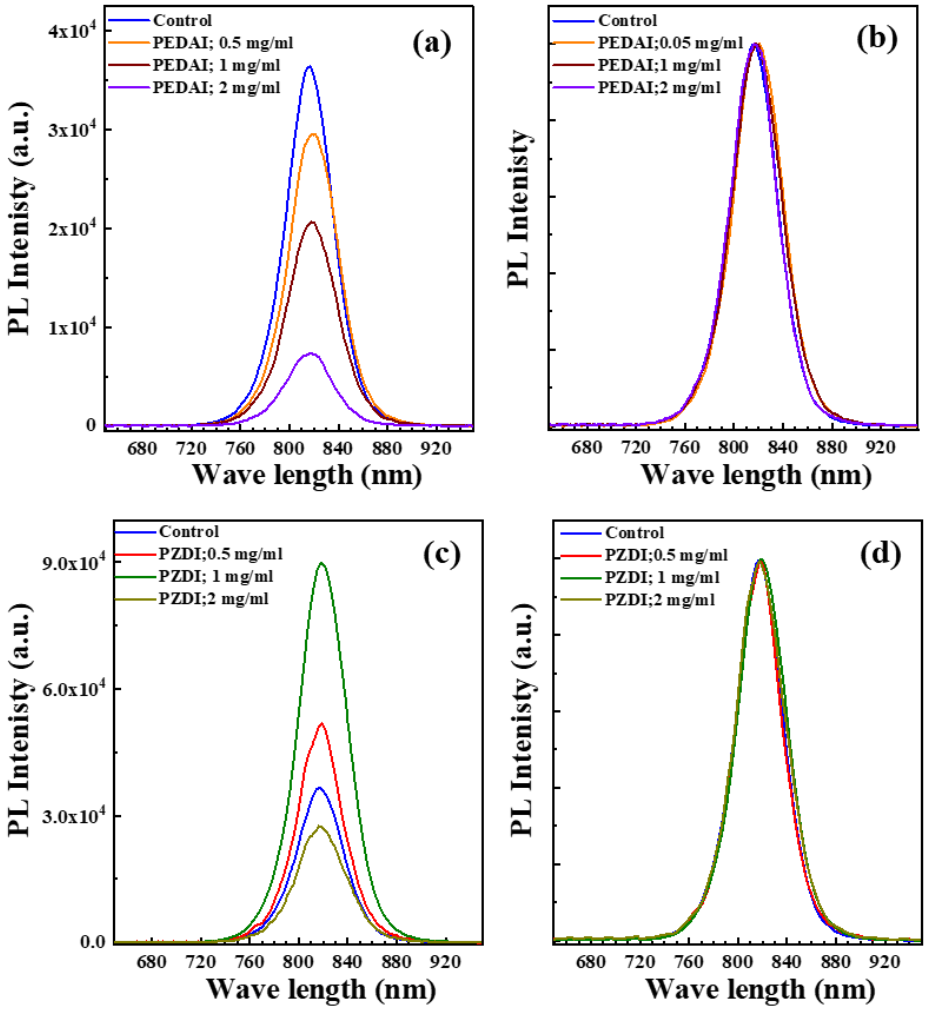


**Fig. S2.** PL spectra of HP films without and with DIM treatments: a) PEDAI or c) PZDI solutions in IPA; 0 (control), 0.5, 1, and 2 mg/ml. b, d) represent normalized PL spectra of respective films.

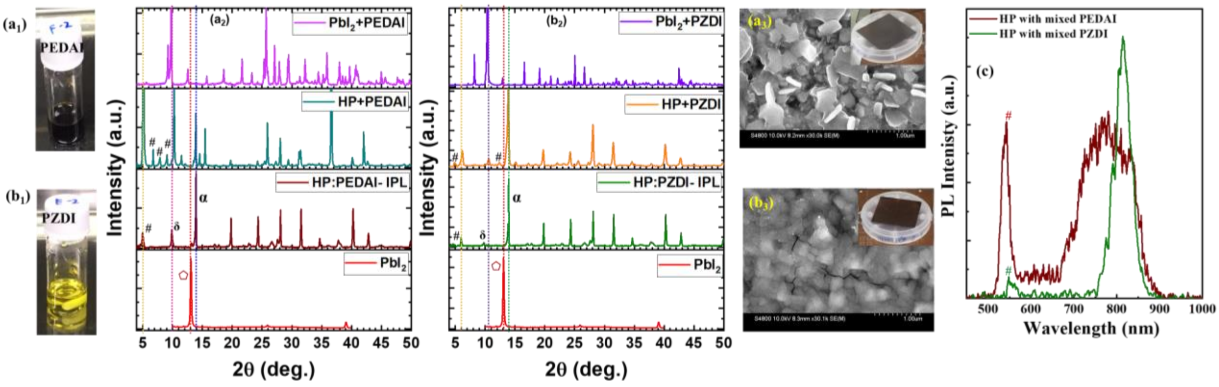


**Fig. S3.** Photo of mixed HP precursor [ $a_1$) PEDAI or $b_1$) PZDI and HP/mixed precursor]. $a_2$, $b_2$) XRD patterns of $PbI_2$ film, HP film surface treated with PEDAI or PZDI (dissolved 2 mg/ml in IPA), HP film with mixed PEDAI or PZDI, and powder crystal prepared by mixing $PbI_2$ and PEDAI or PZDI in 1:2 ratio. Here, # -2D phase with PEDAI or PZDI, ⬠- $PbI_2$ peak, $\delta$- non-photoactive perovskite phase, α-photoactive perovskite phase. $a_3$, $b_3$) SEM images of HP film (mixed precursor: PEDAI or PZDI/perovskite-mixed precursor. c) PL spectra of the HP film prepared using mixed precursor. The shoulder response marked with # in PL spectra stems from the 2D phase formed with PEDAI or PZDI. Note that mixed precursor was prepared by mixing 0.5 M-PEDAI or PZDI + 0.5 M $PbI_2$ and 1M of control precursor ($FA_{0.84}$ $Rb_{0.04}Cs_{0.12}PbI_3$).

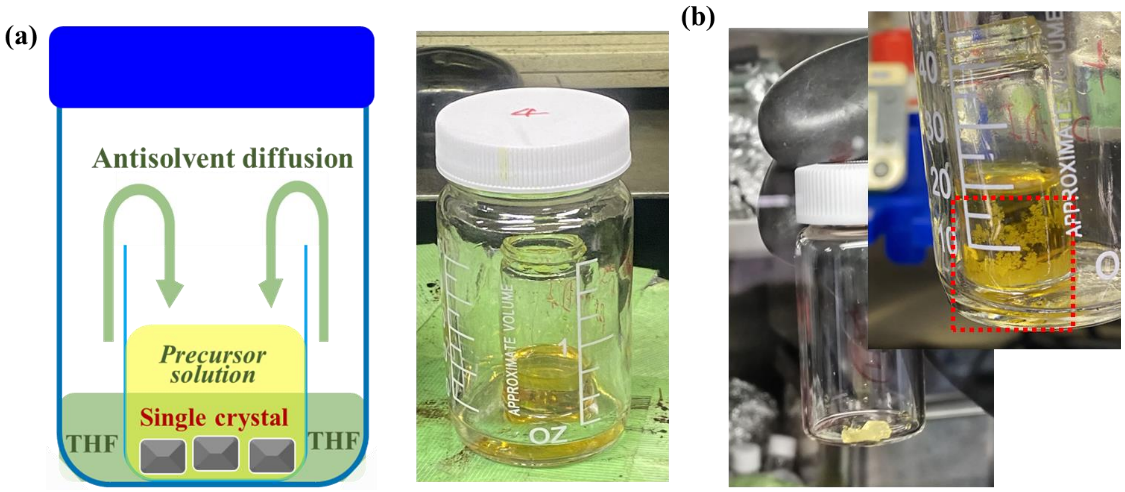


**Fig. S4.** a) Schematic of synthesis of single crystal by antisolvent vapor-assisted crystallization method. of single crystal sample. b) the vial containing precursor solution was sealed in a bottle and crystal growth (displayed in the rectangle) and crystal separated from the vial.

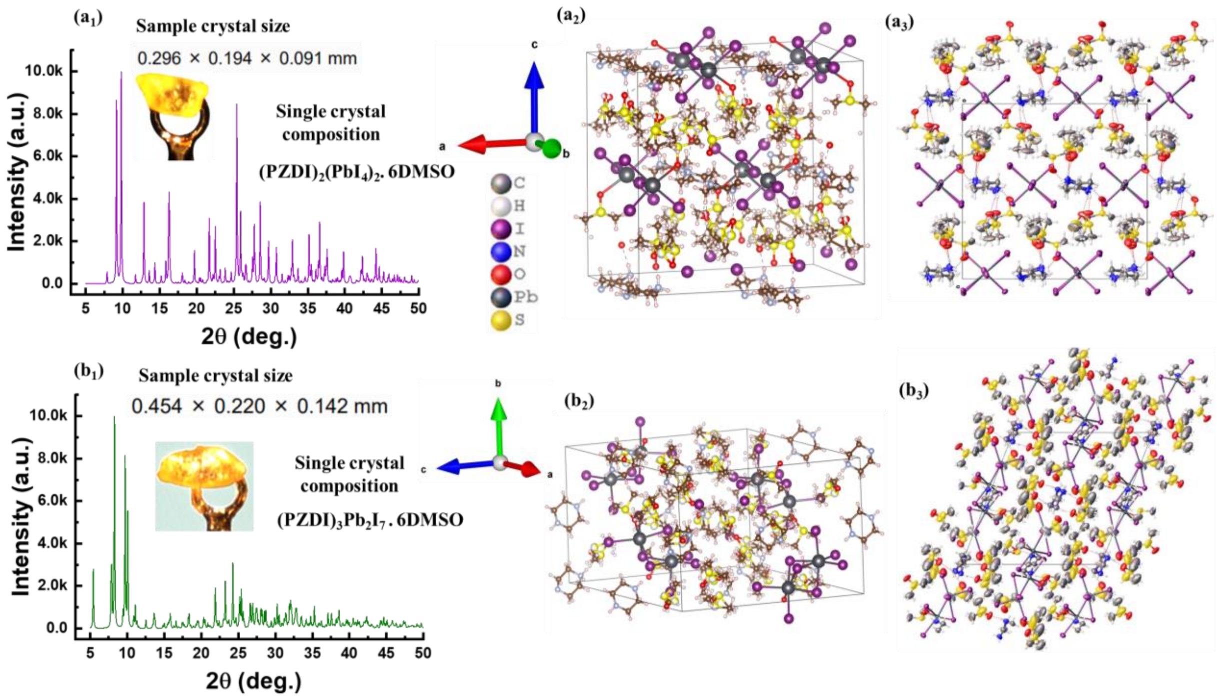


**Fig. S5.** Analysis of a single crystal obtained by adopting the method as depicted in **Fig. S4**. $a_1$) simulated PXRD result of crystal (insets are optical image, dimension, and composition: (Single-crystal structure of PZDI)$_2$(PbI$_4$)$_2$. 6DMSO (CCDC 2311444)) grown in precursor solution (mixing PZDI and $PbI_2$ in 2:1 molar ratio), $a_2$) simulated crystal unit, $a_3$) Packing diagram. $b_1$-$b_3$) corresponding results and properties of the single-crystal structure of PZDI)$_3$Pb$_2$I$_7$. 6DMSO (CCDC 2311446) as grown in precursor solution (mixing PZDI and $PbI_2$ in 1:1 molar ratio).

**Table S1.** Crystal data and refinement of a corresponding single crystal ($(PZDI)_2(PbI_4)_2$. 6DMSO: CCDC 2311444 and $(PZDI)_3Pb_2I_7$. 6DMSO: CCDC 2311446).

| Crystal data | XRD- patterns- Fig. S5a1 | XRD- patterns- Fig. S5b1 |
|---|---|---|
| CIF file | CCDC 2311444 | CCDC 2311446 |
| Chemical Formula | $(PZDI)_2(PbI_4)_2$. 6 DMSO | $(PZDI)_3Pb_2I_7$. 6 DMSO |
| Formula weigh | 1037.33 | 1903.68 |
| Crystal size (mm) | 0.354 × 0.192 × 0.089 | 0.45 × 0.22 × 0.14 |
| ***μ*** ($mm^{-1}$) | 11.055 | 10.968 |
| Radiation type | Mo Kα | Mo Kα |
| Wavelength (Å) | 0.71073 | 0.71073 |
| Temperature | 293(2) | 293.00(10) |
| Crystal system | Orthorhombic | Monoclinic |
| Space group | Pna21 | P 21 /n |
| a, b, c (Å) | 20.3941 (3), 13.7425 (2), 19.2851 (3) | 19.4940(3), 14.11900(10), 20.1039(2) |
| a, b, g (°) | 90, 90,90 | 90, 111.049(2), 90 |
| V ($Å^3$ ) | 5404.96 (14) | 5164.09(12) |
| Z | 8 | 4 |
| | Data collection | |
| No. of measured | 134993 | 170497 |
| No. of independent | 14685 | 14425 |
| Completeness | 1 | 0.876 |
| $R_{int}$ | 0.0498 | 0.0613 |
| 2θmax (°) | 61.818 | 61.998 |
| | Refinement | |
| $R_1$ [$F^2$ > 2s ($F^2$ )] | 0.0439 | 0.0395 |
| wR(F2 ) | 0.0978 | 0.0836 |
| S | 1.149 | 1.167 |
| No. of reflections | 14685 | 14425 |
| No. of parameters | 475 | 435 |

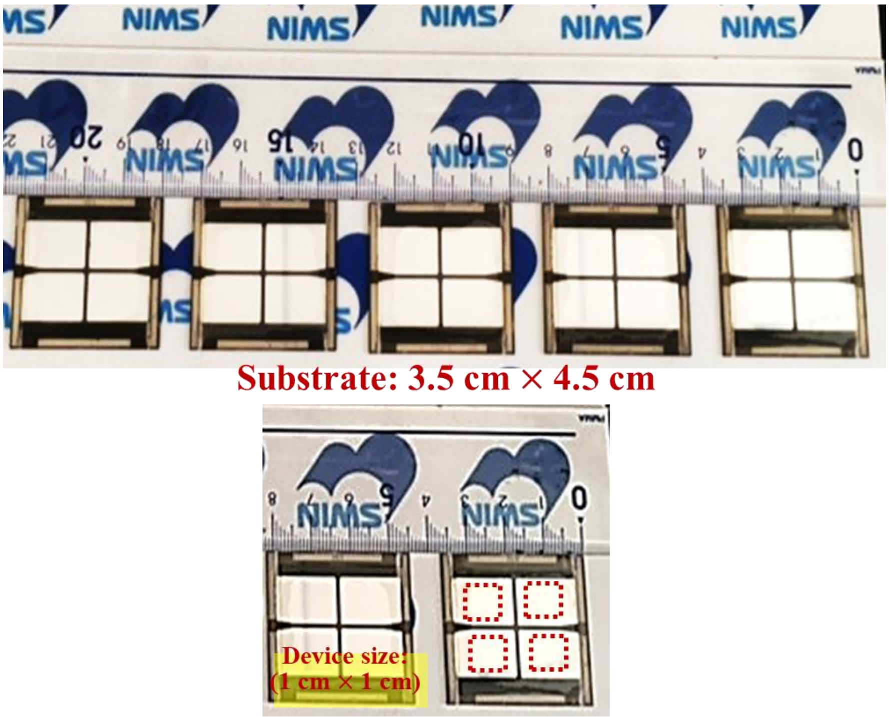


**Fig. S6.** Photographs of a set of HHPSC devices prepared on ITO substrate (4.5 cm ×3.5 cm) and scale of each device size (1cm×1cm) used for measurement mask.

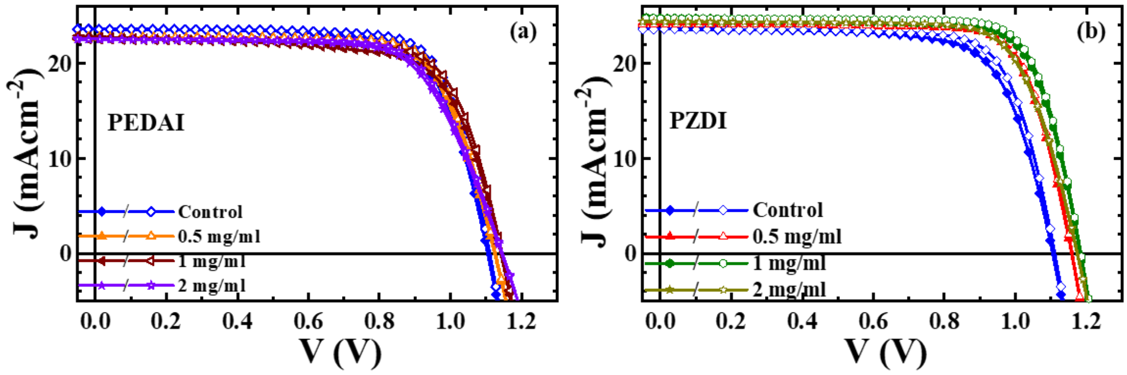


**Fig. S7.** *J-V* curves of the control and DIM; a) PEDAI or b) PZDI treated HPSCs. ▼/▽ forward /reverse scan direction.

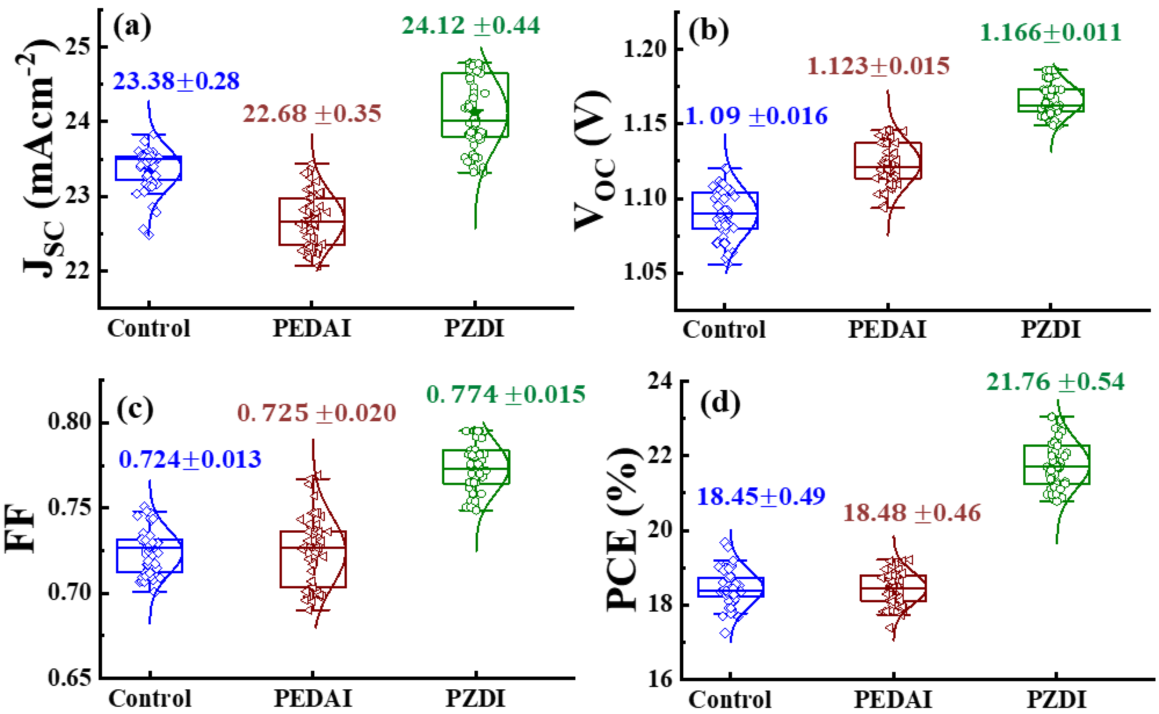


**Fig. S8.** Statistics of PV characteristic parameters of control and PEDAI or PZDI-treated HPSCs, including; a) $V_{OC}$, b) $J_{SC}$, c) *FF*, and d) *PCE*. The data shown above the box distribution corresponds average and standard deviation for corresponding device parameters. These data consist of 50 devices from 6 batches.

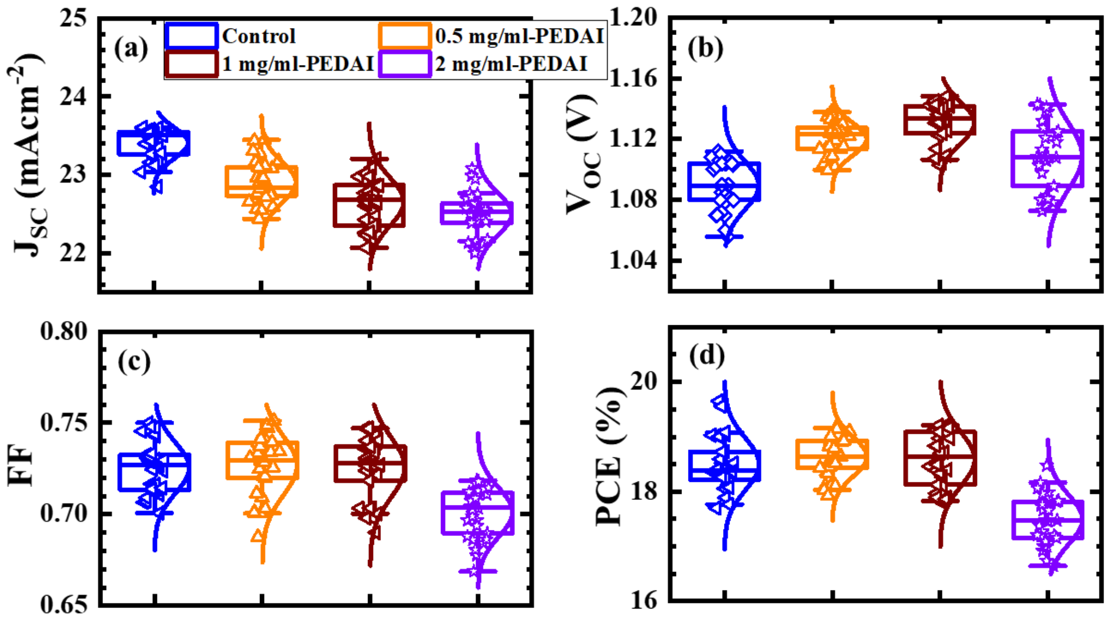


**Fig. S9.** Statistics of PV characteristic parameters of control and PEDAI-treated HPSCs, including; a) $V_{OC}$, b) $J_{SC}$, c) *FF*, and d) *PCE*. These data consist of 30 devices from 4 batches.

**Table S2.** Photovoltaic parameters of the best-performing HPSCs with MA-free HP (without and with PEDAI surface treatment). The parameters given inside the parentheses represent the average values and standard deviation.

| Device/parameters | Control | 0.5 mg/ml | 1 mg/ml | 2 mg/ml |
|---|---|---|---|---|
| $J_{SC}$ (mAcm$^{-2}$) | 23.56 (23.41 ±0.21) | 22.75 (22.91±0.26) | 22.73 (22.66±0.31) | 22.67 (23.51±0.27) |
| $V_{OC}$ (V) | 1.112 (1.089 ±0.016) | 1.132 (1.118 ±0.014) | 1.145 (1.131 ±0.013) | 1.142 (1.114 ±0.022) |
| *FF* | 0.750 (0.725 ±0.014) | 0.745 (0.726 ±0.016) | 0.738 (0.725 ±0.016) | 0.702 (0.701 ±0.013) |
| *PCE* (%) | 19.64 (18.51 ±0.48) | 19.18 (18.61 ±0.394) | 19.22 (18.89 ±0.48) | 18.17 (17.49 ±0.46) |

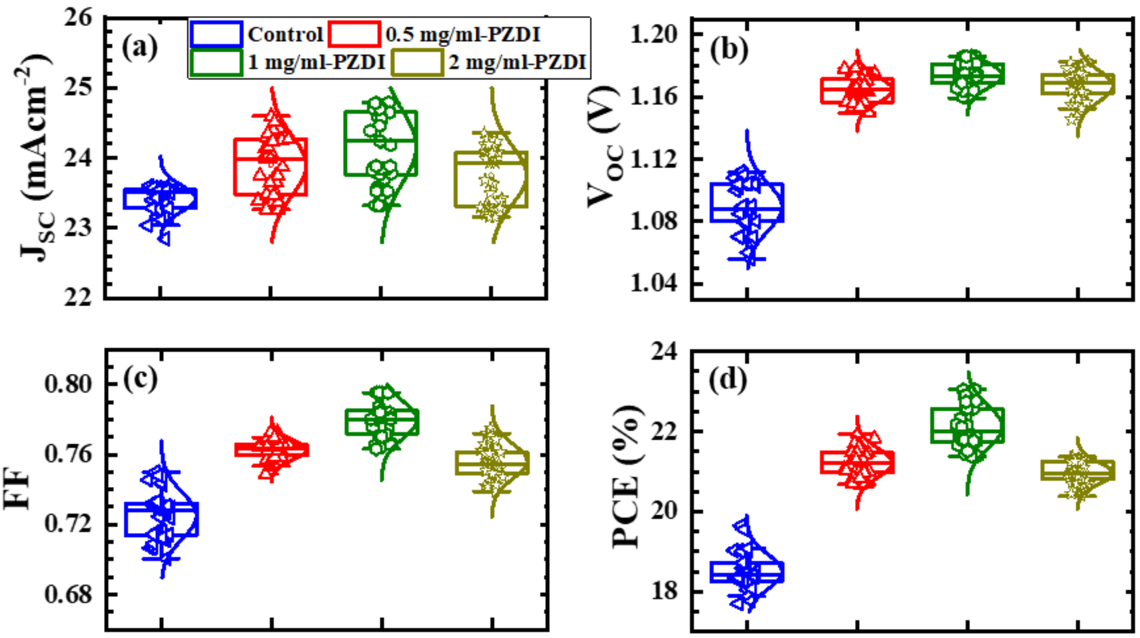


**Fig. S10.** Statistics of PV characteristic parameters of control and PZDI treated HPSCs, including; a) $V_{OC}$, b) $J_{SC}$, c) *FF*, and d) *PCE*. These data consist of 30 devices from 4 batches.

**Table S3.** Photovoltaic parameters of the best-performing HPSCs with MA-free HP (without and with PZDI surface treatment). The parameters given inside the parentheses represent the average values and standard deviation.

| Device/parameters | Control | 0.5 mg/ml | 1 mg/ml | 2 mg/ml |
|---|---|---|---|---|
| $J_{SC}$ (mAcm$^{-2}$) | 23.56 (23.41 $\pm$0.21) | 24.63 (23.97 $\pm$0.42) | 24.79 (24.15 $\pm$0.51) | 24.54 (23.71 $\pm$0.43) |
| $V_{OC}$ (V) | 1.112 (1.089 $\pm$0.016) | 1.181 (1.663 $\pm$0.009) | 1.186 (1.176 $\pm$0.008) | 1.174 (1.168 $\pm$0.011) |
| *FF* | 0.750 (0.725 $\pm$0.014) | 0.765 (0.762 $\pm$0.006) | 0.784 (0.775 $\pm$0.012) | 0.756 (0.752 $\pm$0.013) |
| *PCE* (%) | 19.64 (18.51 $\pm$0.48) | 22.25 (21.27 $\pm$0.39) | 23.05 (22.35 $\pm$0.54) | 21.78 (20.95 $\pm$0.49) |

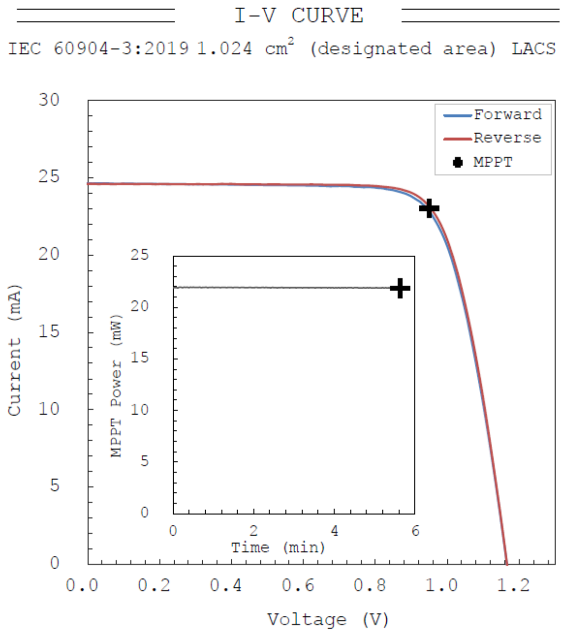


Date :13 Jun 2022
Sample No. :NIMS_FAPVK-5
Repeat Times :1

| Scan Mode | | Forward | Reverse | MPPT+ |
|---|---|---|---|---|
| $I_{sc}$ | (mA) | 24.65 | 24.61 | - |
| $V_{oc}$ | (V) | 1.166 | 1.166 | - |
| $P_{max}$ | (mW) | 21.72 | 22.00 | **21.88** |
| $I_{pmax}$ | (mA) | 23.15 | 23.30 | **23.04** |
| $V_{pmax}$ | (V) | 0.938 | 0.944 | **0.950** |
| F.F. | (%) | 75.6 | 76.6 | - |
| Eff(da) | (%) | 21.20 | 21.47 | **21.36** |
| DTemp. | (°C) | 25.0 | 25.0 | **25.0** |
| MTemp. | (°C) | 25.1 | 25.1 | **25.1** |
| Dirr. | (mW/cm²) | 100.0 | 100.0 | **100.0** |
| Mirr. | (mW/cm²) | 99.9 | 99.9 | **99.9** |

Ref.Device No. CSI03+KG3-2t
Cal.Val.of Ref. 60.22 (mA at 100mW/cm²)
**+: After MPPT 5.7 min**
**Total Illumination Time : 10 min**

AIST

**Fig. S11.** Certified results from an accredited photovoltaic certification laboratory (AIST, Japan). The certified PCE is 21.36% - certified aperture with an area of ~1.024 cm$^2$. The certified J-V curves with double scanning give PCE forward: 21.20% and PCE reverse: 21.47%. (HP film: $Rb_{0.4}Cs_{0.12}FA_{0.84}PbI_3$ with PZDI surface treatment).

**Table S4.** Summary of certified/record device large area (1 $cm^2$) reports (Pb-HP using multiple cations, anions, functional additives, and interfacial layer).

| Device Type | Device Structure | Perovskite | Additive | Area ($cm^2$) | PCE (%) | Reported Efficiency | date | Ref. |
|---|---|---|---|---|---|---|---|---|
| Regular (n-i-p) | ITO/c-$TiO_2$/$TiO_2$ nanorods/PMMA:PCBM/**Perovskite/**PMMA/Spiro-OMeTAD)/MoOx/IZO/**Au** | (Cs,FA,MA)Pb(I,Br)$_3$ | ----- | 1.0 | 21.6 | Certified | 2021 | 1 |
| Regular (n-i-p) | FTO/$TiO_xN_y$/meso-$TiO_2$/PMMA: PCBM/**perovskite/**PMMA/$P_3$HT: CuPc/**Au** | (Cs,FA,MA)Pb(I,Br)$_3$ | 0.01 M-$PbCl_2$/MACl | 1.0 | 22.6 | Certified | 2022 | 2 |
| Regular (n-i-p) | FTO/SnO2/**perovskite/**spiro-OMeTAD/EVA/**Cu-Ni-Graphene** | FAMAPb(I,Br)3 | ----- | 0.09 | 24.37 | Certified | 2022 | 3 |
| | | | | 1.02 | 20.76 | | | |
| Inverted (p-i-n) | ITO/NiOx-nanoparticles/(IL)EMDP/**Perovskite/**PCBM/BCP/**Au** | (Cs,FA,MA)Pb(I,Br)$_3$ | ----- | 1.01 | 20.91 | Not certified | 2021 | 4 |
| Inverted (p-i-n) | ITO/PTAA/PIC-$Al_2O_3$/**Perovskite/**C60/BCP/**Ag** | (FA,MA)CsPb(I,Br)$_3$ | MACl | 0.06 | 24.9 | certified | 2023 | 5 |
| | | | | 1.06 | 23.30 | | | |
| Regular (n-i-p) | ITO/$SnO_2$/perovskite/Spiro-OMeTAD/Au | (Cs,FA)$PbI_3$ | 4-MeO-PEAI | 1.00 | 23.7 | certified | 2022 | 6 |
| Regular (n-i-p) | FTO/$SnO_2$/perovskite/Spiro-OMeTAD/ Au | (Cs,FA,MA)Pb(I,Br,Cl)$_3$ | 2-Cl-PEAI | 0.1 | 23.9 | certified | 2023 | 7 |
| | | | | 1.00 | 23.7 | | | |
| Regular (n-i-p) | FTO/$SnO_2$(ALD)/PCBM/PMMA/**Perovskite/**PMMA/Spiro/**Au** | (Rb,Cs,FA)$PbI_3$ | ----- | 0.1024 | 20.35 | Not certified | 2018 | 8 |
| Regular (n-i-p) | FTO/$TiO_2$/perovskite/ Spiro-OMeTAD/ Au | (Cs,FA)$PbI_3$ | CoFAc | 0.09 | 24.64 | Not certified | 2023 | 9 |
| Inverted (p-i-n) | ITO/2PACz/**Perovskite/PEACl/**$C_{60}$/BCP/**Au** | (Cs,FA)$PbI_3$ | PEACl | 0.123 | 22.3 | Not Certified | 2021 | 10 |
| Inverted (p-i-n) | ITO/NiOx-iink/**Perovskite/**$C_{60}$/BCP/**Cu** | (Cs,FA,)Pb(I,SCN)$_3$ | TCMAI-doping | 0.04 | 23.2 | Certified | 2023 | 11 |
| Inverted (p-i-n) | ITO/NiOx-sputtered/MeO/**Perovskite/**PZDI/$C_{60}$/BCP/**Ag** | (Rb,Cs,FA)$PbI_3$ | PZDI | 1.024 | 21.47 (23.17) | Certified | *This work (Record certify PCE for inverted device structure) | |

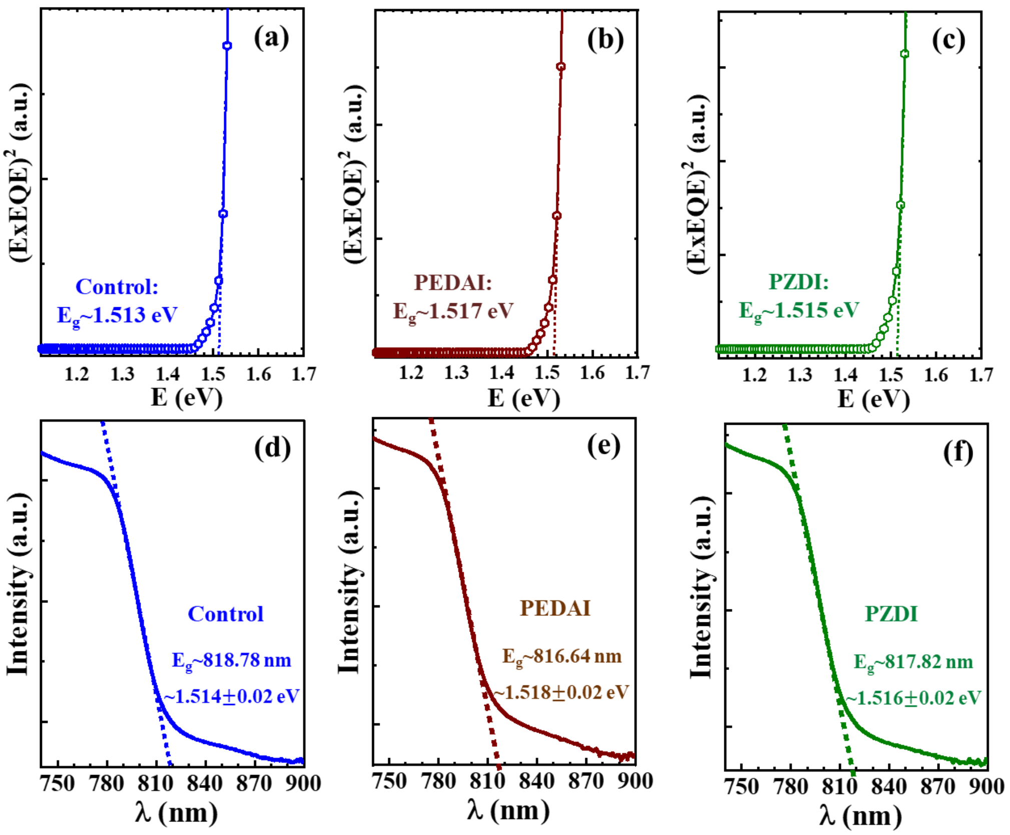


**Fig. S12.** Estimation of bandgap energy ($E_g$) of HP layers (control and with surface treatment (PEDAI or PZDI)). a-c) $E_g$ estimated from *EQE* analysis. d-f) $E_g$ calculated from absorption spectra of respective films.

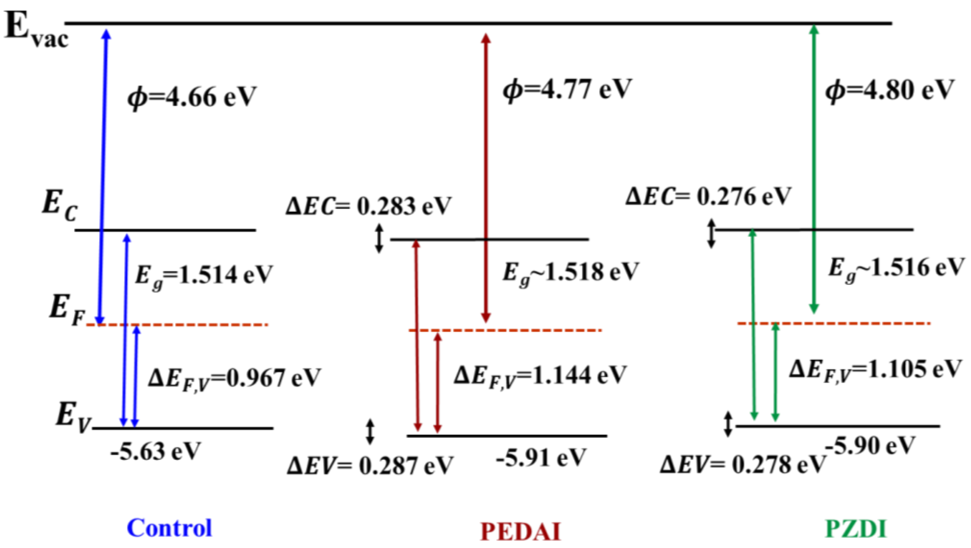


**Fig. S13.** Energy band schematics of control and PEDAI or PZDI treated film extracted from UPS spectra. Note that $E_g$ for this schematic is calculated from absorption spectra ( s Fig. S12d-f).

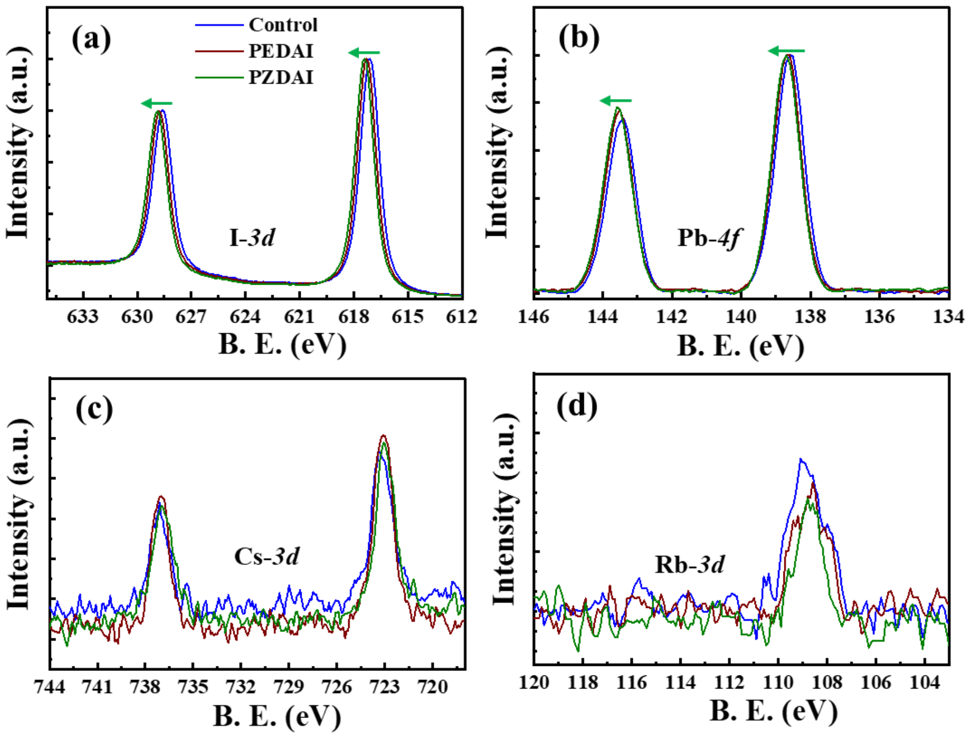


**Fig. S14.** XPS-spectra for the control and PEDAI or PZDI treated films; a) I-*3d* core, b) Pb-*4f*, c) Cs-*3d* core, and d) Rb-*3d* core. The arrowhead indicates the shifting direction of the XPS Characteristic peak.

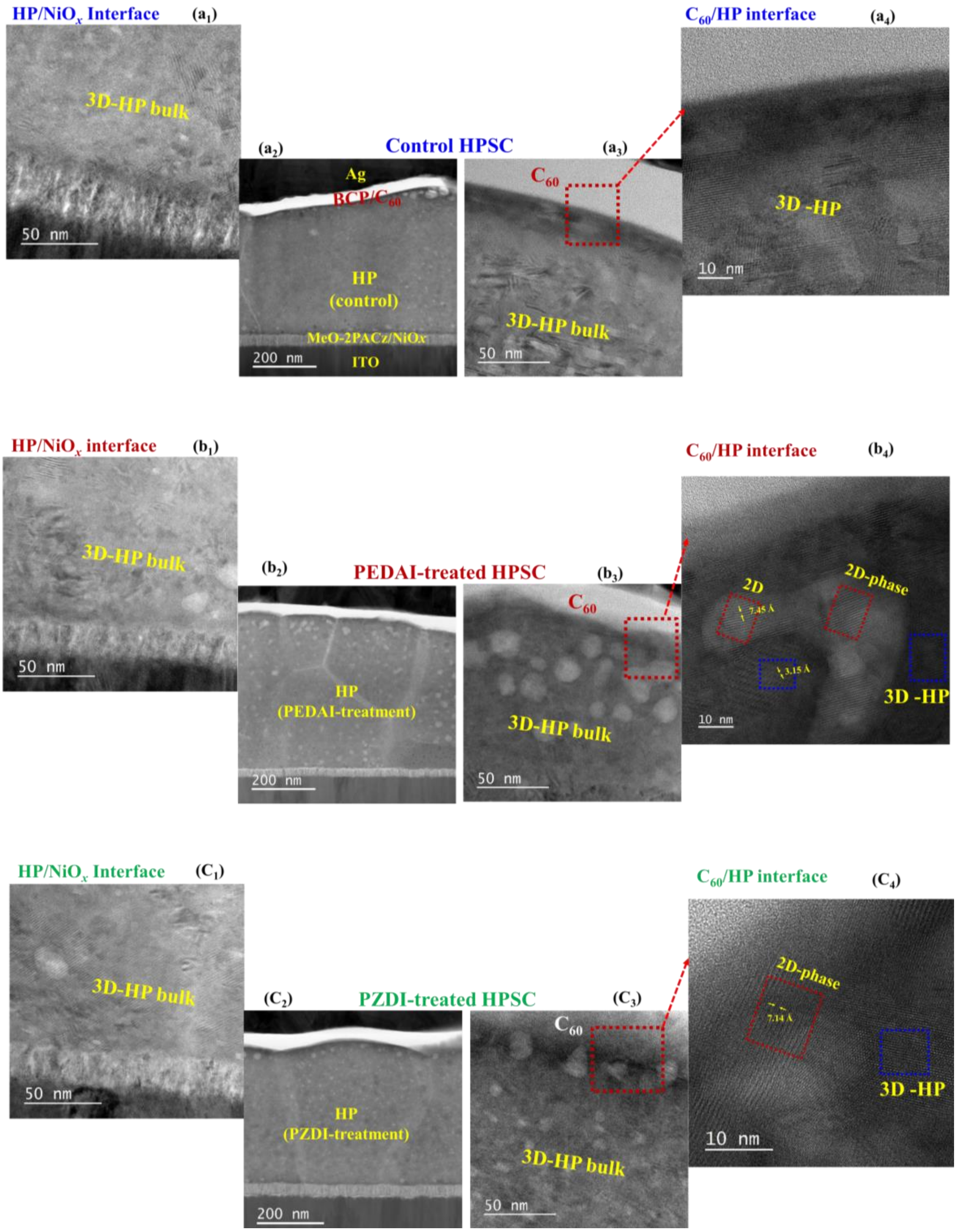


**Fig. S15.** Cross-sectional HR-TEM image of HPSCs for interface analysis; control ($a_1$-$a_4$), PEDAI-treated ($b_1$-$b_4$), and PZDI-treated ($c_1$-$c_4$). HP/$NiO_x$ interface ($a_1$,$b_1$,$c_1$)), over all cross-section ($a_2$,$b_2$,$c_2$), $C_{60}$/HP interface (($a_3$,$b_3$,$c_3$), and interface of $C_{60}$/HP top surface ($a_4$,$b_4$,$c_4$). Note that the surface passivation with PEDAI forms a rich 2D phase at the interface and grain boundaries. On the other hand, PZDI surface treatment grows with an evenly distributed 2D phase interface on the surface of 3D-HP with diffusion through grain boundaries to some extent.

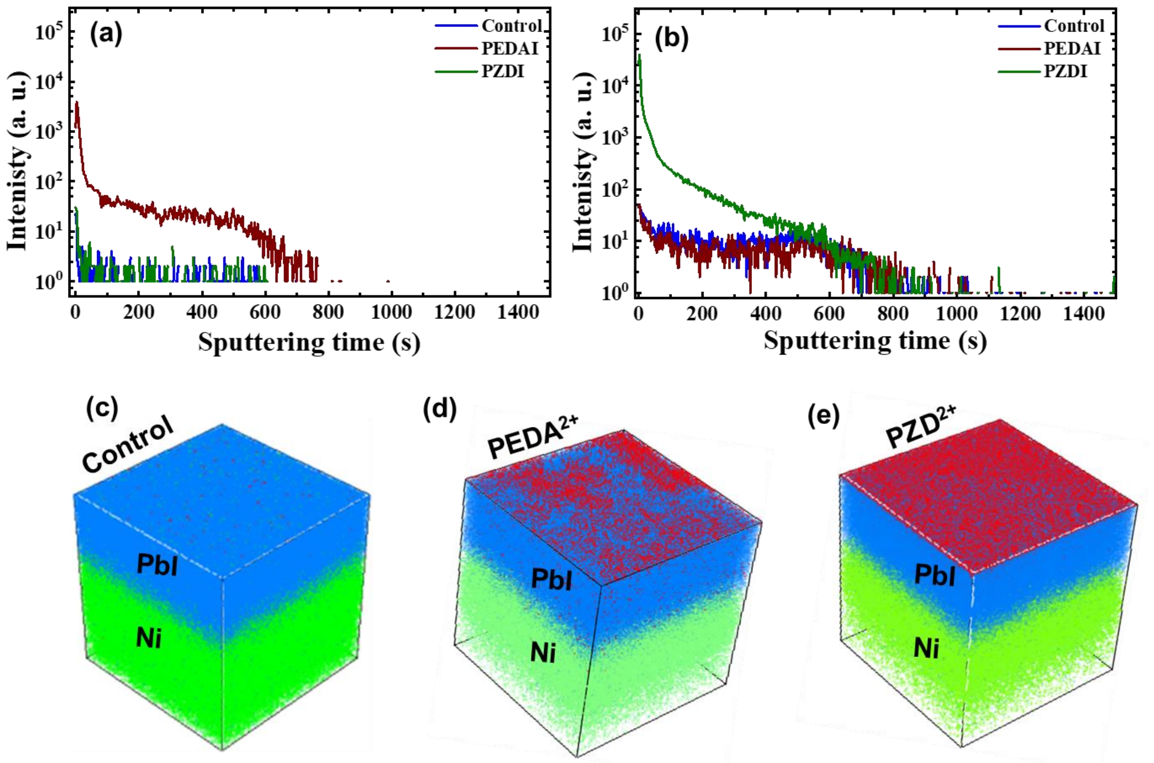


**Fig. S16.** ToF-SIMS depth profiles of control and surface-treated HP film. a) $PEDA^{2+}$ distribution and b) $PZD^{2+}$ distribution in control and DIM-treated HP films. Reconstructed 3D maps; Distributions of passivated molecules in HP film c) control, d) PEDAI-treated, and e) PZDI-treated.

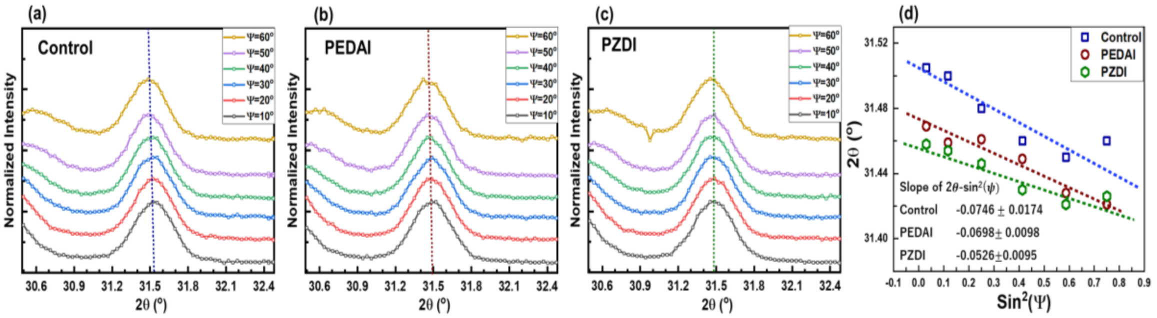


**Fig. S17.** XRD spectrum at different tilt angles for a) control, b) PEDAI, and c) PZDI devices, respectively. d) Residual strain extracted from the corresponding HPSC device' diffraction strain data as a function of $\sin^2\psi$.

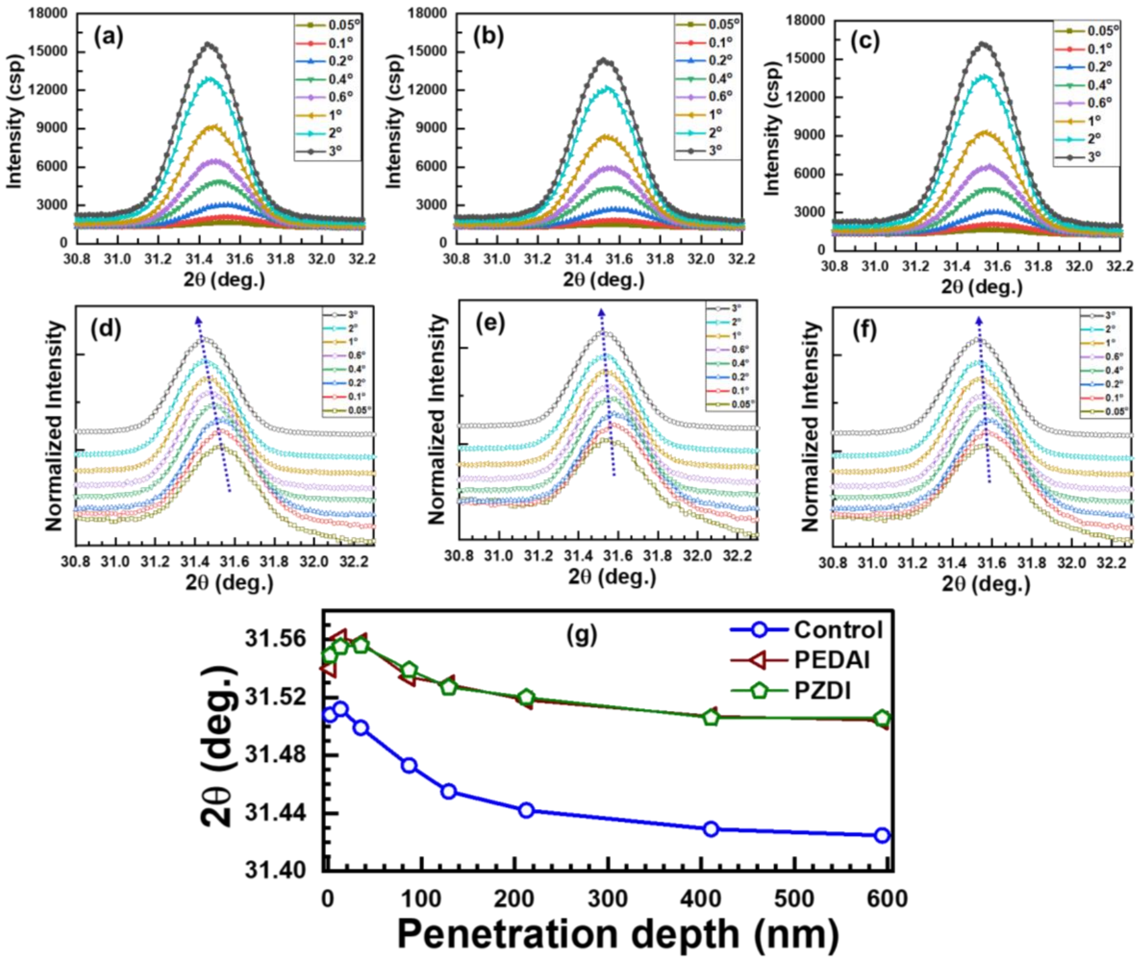


**Fig. S18.** GIXRD spectra: a, d) control, b, e) PEDAI, and c, f) PZDI.  g) plot of penetration depth corresponds to the grazing incident angle. X-ray attenuation length (penetration depth) into the perovskite film (estimated density ~3.86 $gcm^{-3}$) was calculated using a report by Davis and co-workers ( Atomic Data and Nuclear Data Tables, 1993, 54, 181-342) and Rigaku-manual.

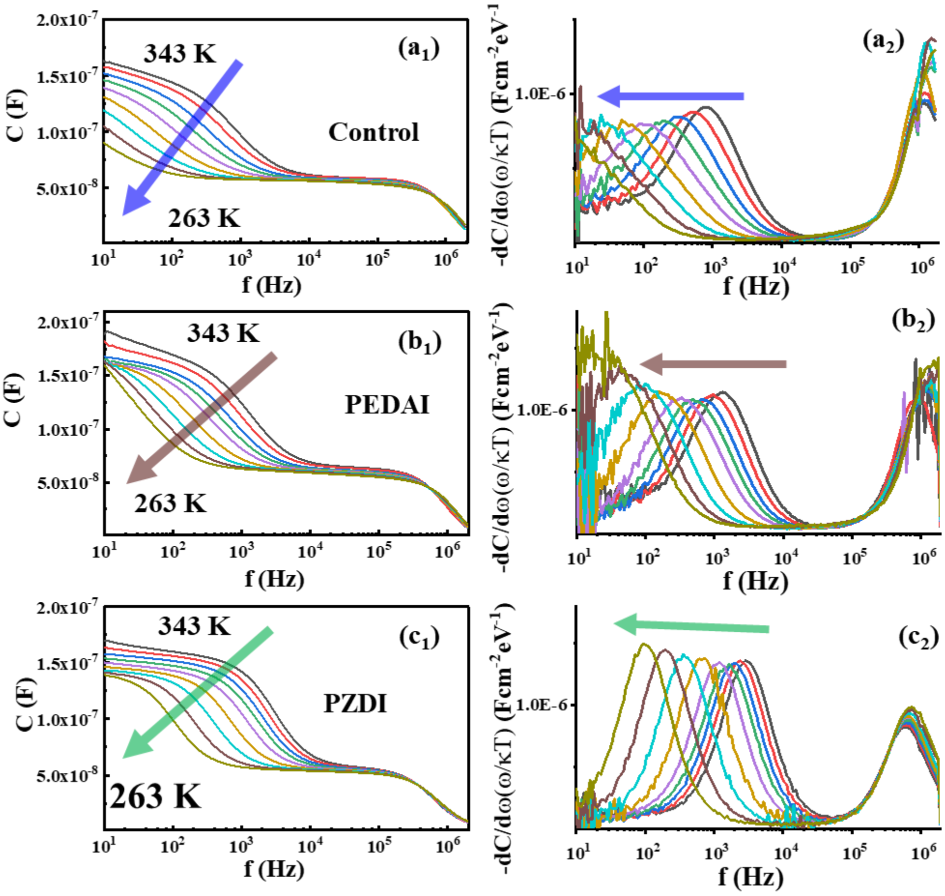


**Fig. S19.** Thermal admittance spectra (*TAS*). $a_1$-$c_1$) *C*-*f*-*T* spectra of control and PEDAI or PZDI treated devices. $a_2$-$c_2$) differentiation of respective *C*-*f*-*T* spectra for determination of inflection frequencies.

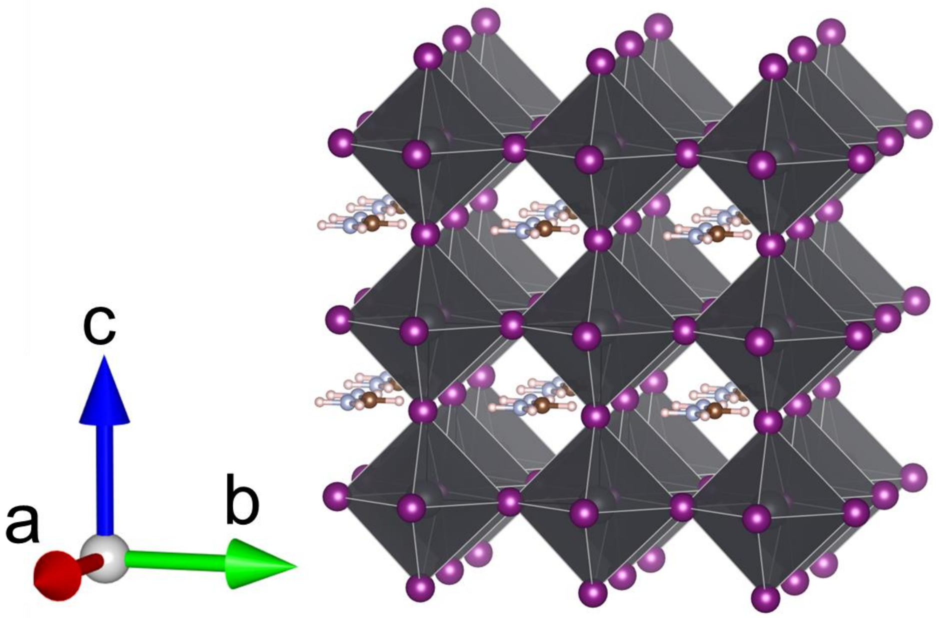


**Fig. S20**. The optimized pseudo-cubic structure of the bulk $FAPbI_3$. Pb atoms are colored grey, I atoms are purple, C atoms are brown, N atoms are light grey, and H atoms are light pink. The orientation of the lattice axes (a/b/c and arrows) is shown in the insert. Figure was created using VESTA software.[12]

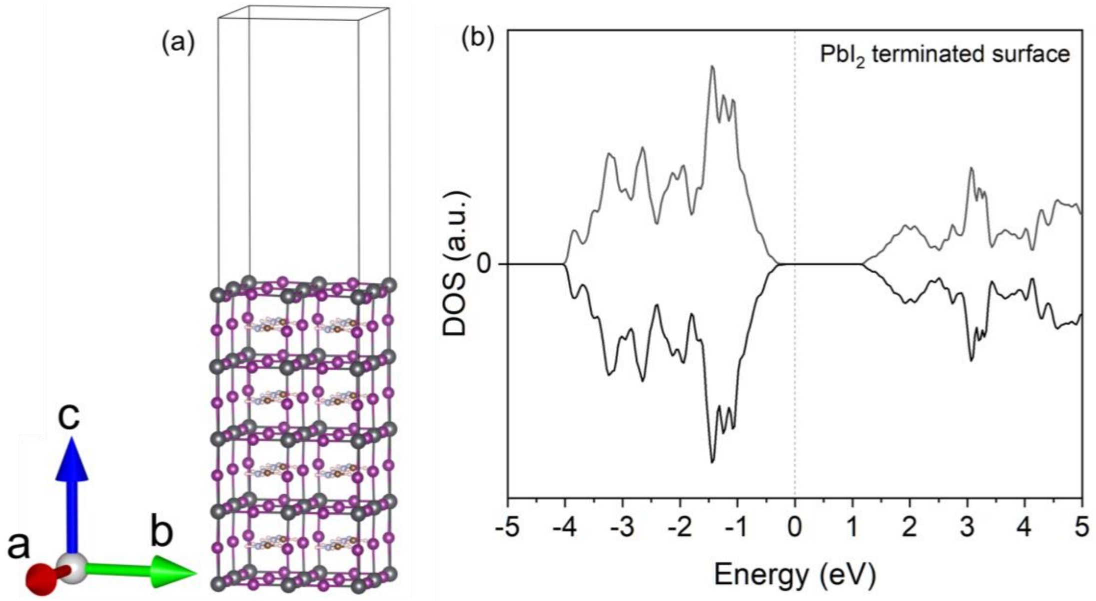


**Fig. S21**. a) The optimized structure of the 2x2 unit cell of $PbI_2$-terminated surface (001) of the pseudo-cubic $FAPbI_3$. b) Total density of states calculated for the defect-free $PbI_2$-terminated surface of $FAPbI_3$. The orientation of the lattice axes (a/b/c and arrows) is shown in the insert. Figure in (a) was created using VESTA software.[12]

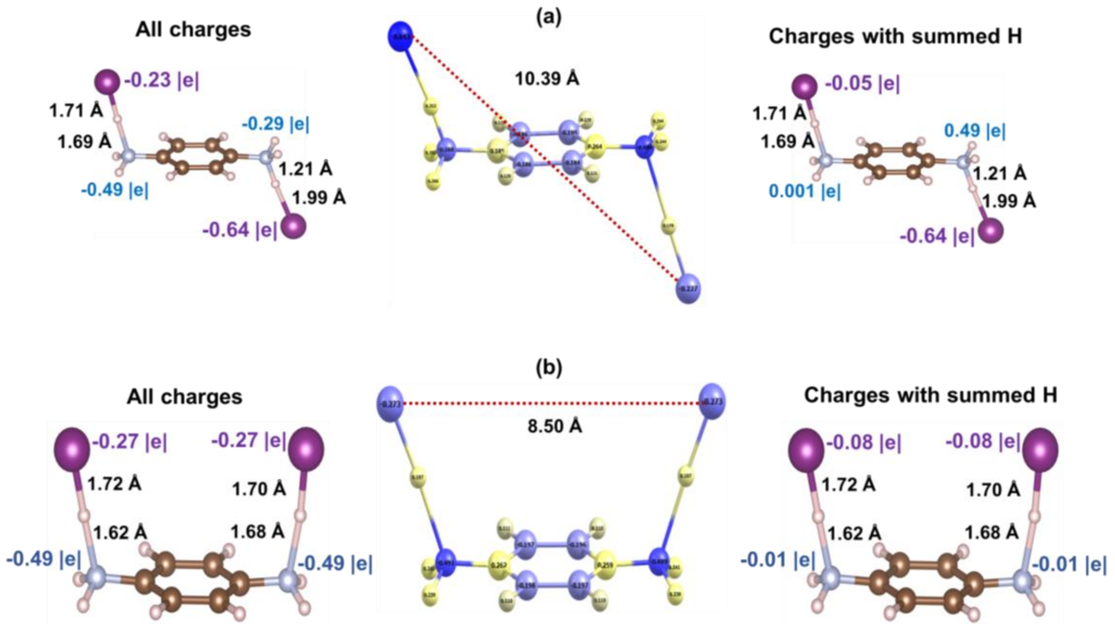


**Fig. S22**. The optimized structures of a free 1,4-phenylenediamine dihydriodide (PEDAI), $C_6H_8N_2$*2HI, molecule. (a) The most stable *trans*-isomer structure, and (b) the low-energy *cis*-isomer form. Mulliken charges or Mulliken charges with summed H (iodine and nitrogen atoms) were calculated at the B3LYP/def2TZVP level of theory with the use of Gaussian 09.[17]

Free 1,4-phenylenediamine dihydriodide (PEDAI), $C_6H_8N_2$*2HI, molecules possess several isomeric forms depending on the position of the HI compounds. The most stable *trans*-isomer is shown in Figure S18a, while its *cis* form (Fig. S22b) is only 0.086 eV less stable. When PEDAI adsorbs on the $PbI_2$-terminated surface of $FAPbI_3$ adsorption of the *cis*-form became energetically favorable, as it maximizes interaction of I atoms of PEDAI with the surface Pb atoms.

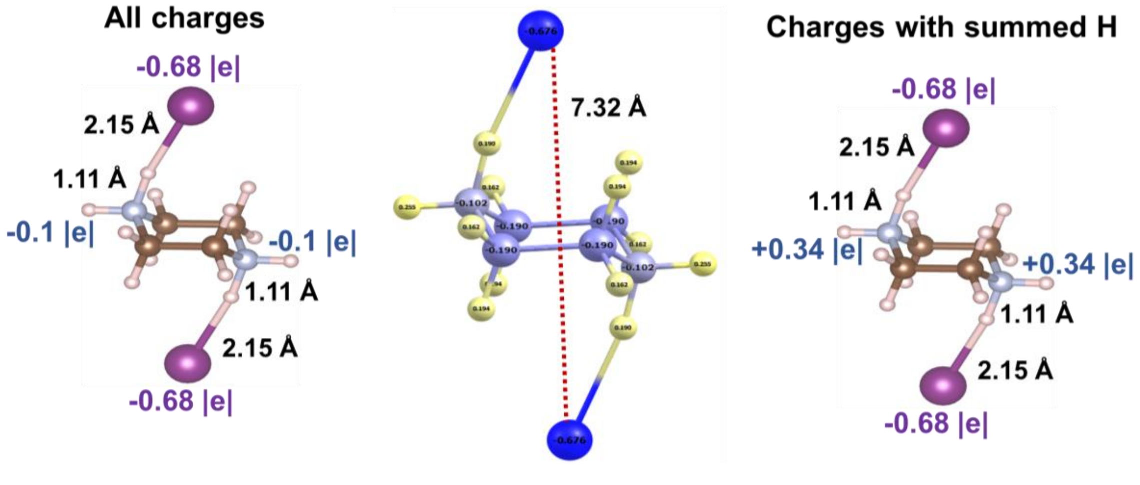


**Fig. S23**. The optimized structure of a free piperazine dihydriodide (PZDI), $C_4H_{10}N_2$*2HI, molecule. Mulliken charges were calculated at the B3LYP/def2TZVP level of theory with the use of Gaussian 09.[18]

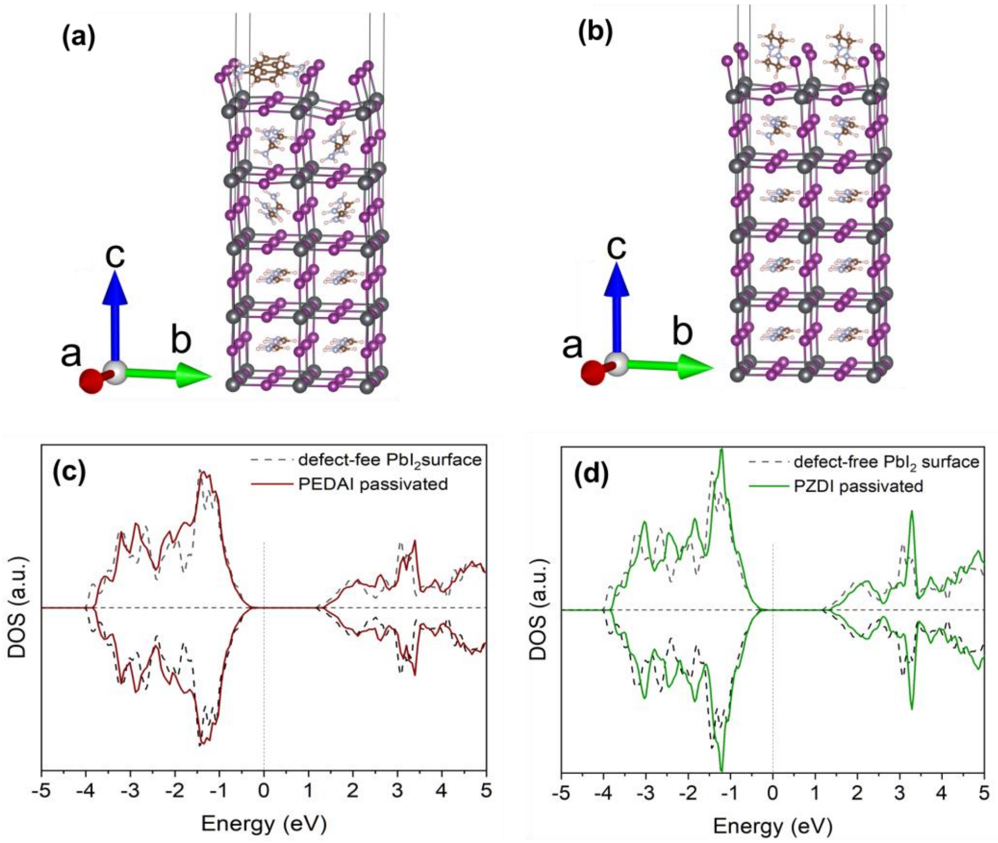


**Fig. S24.** Optimized structures of a) PEDAI and b) PZDI adsorbed on the defect-free $PbI_2$-terminated surface of $FAPbI_3$. The spin-polarized total DOS calculated for the $PbI_2$-terminated surface of $FAPbI_3$ covered by c) PEDAI and d) PZDI. The orientation of the lattice axes (a/b/c and arrows) is shown in the insert. Figures in a) and b) were created using VESTA software.[12]

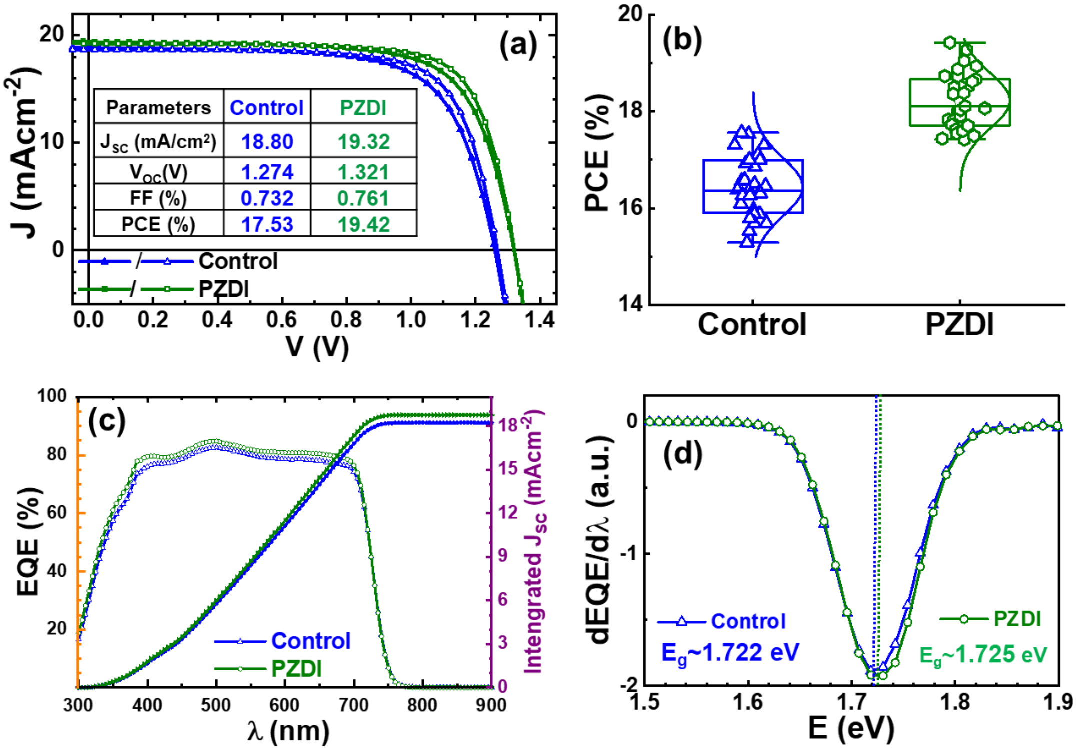


**Fig. S25.** Device characteristics for wide bandgap-HP (Pb-HP; *$FA_{0.84}Cs_{0.12}Rb_{0.04}Pb(I_{0.63}Br_{0.37})_3$) (WB-HP)*. a) *J-V* curves (device parameters, Table S5) (filled/open symbol- forward /reverse scan direction) of control and PZDI-treated HPSCs. b) PCE statistics of devices. c) *EQE* spectra of respective devices. The values of integrated $J_{SC}$ extracted from *EQE* spectra; 18.22 and 18.78 mAcm$^{-2}$. (d) Estimation of bandgap energy ($E_g$) from EQE analysis.

**Table S5.** Photovoltaic parameters of the best-performing WB-HPSCs corresponding to J-V curves (**Fig. S25**). F and R- scan stand for forward and reverse scan directions. The statistical data (control or PZDI treatment) are taken from 30 devices (average (avg) and standard deviation (sd)) from 5 batches.

| Device parameters | *WB-HPSCs* | | | | | |
|---|---|---|---|---|---|---|
| | Control | | | PZDI | | |
| | F-scan | R-scan | statistics (avg±sd) | F-scan | R-scan | Statistics (avg±sd) |
| $J_{SC}$ (mA/cm$^2$) | 18.86 | 18.80 | 18.68 ±0.62 | 19.43 | 19.32 | 19.08 ±0.114 |
| $V_{OC}$ (V) | 1.262 | 1.274 | 1.26 ±0.003 | 1.316 | 1.321 | 1.312 ±0.002 |
| *FF* | 0.695 | 0.732 | 0.709 ±0.004 | 0.724 | 0.761 | 0.724 ±0.017 |
| *PCE* (%) | 16.54 | 17.53 | 16.76 ±0.69 | 18.51 | 19.42 | 18.46 ±0.42 |

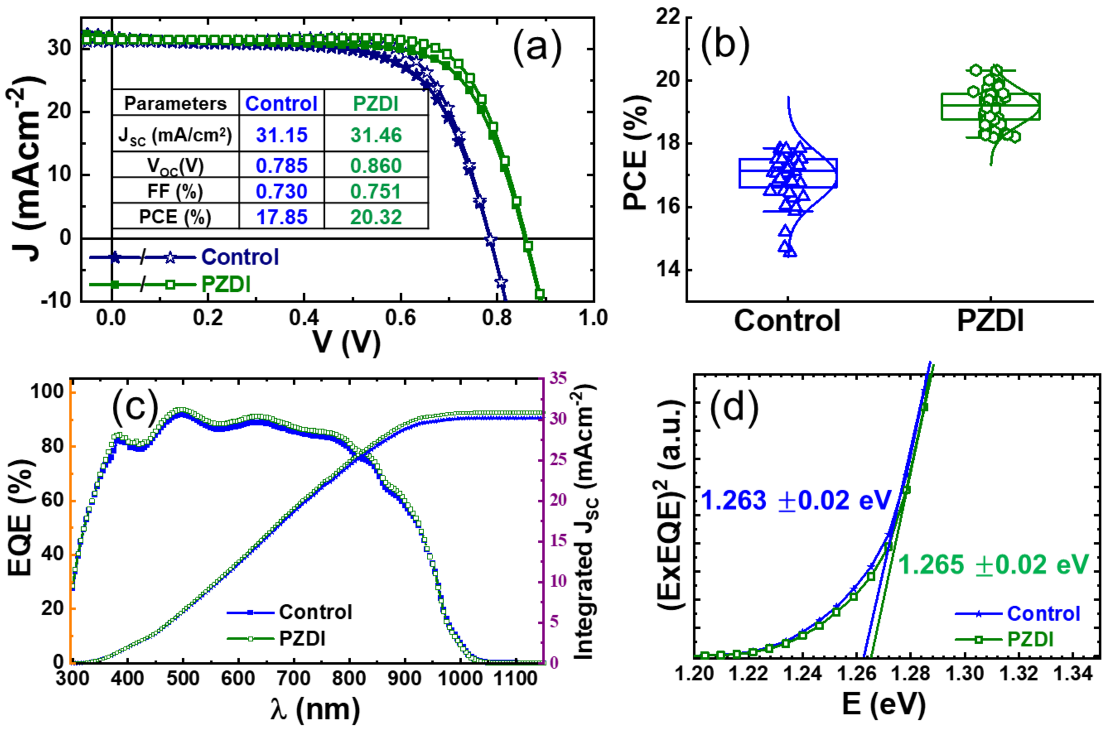


**Fig. S26.** Device characteristics for narrow bandgap-HP (Sn-Pb-HP; $FA_{0.85}MA_{0.1}Cs_{0.05}(Pb_{0.5}Sn_{0.5})I_3)$ *(NB-HP)*. a) *J-V* curves (device parameters, Table S6) (filled/open symbol- forward /reverse scan direction) of control and PZDI-treated HPSCs. b) PCE statistics of devices. c) *EQE* spectra of respective devices. The values of integrated $J_{SC}$ extracted from *EQE* spectra; 30.19 and 30.86 mAcm$^{-2}$. d) Estimation of bandgap energy ($E_g$) from EQE analysis.

**Table S6.** Photovoltaic parameters of the best-performing NB-HPSCs corresponding to J-V curves (**Fig. S26**). F and R- scan stand for forward and reverse scan directions. The statistical data (control or PZDI treatment) are taken from 30 devices (average (avg) and standard deviation (sd)) from 5 batches.

| Device parameters | *NB-HPSCs* | | | | | |
|---|---|---|---|---|---|---|
| | Control | | | PZDI | | |
| | F-scan | R-scan | statistics (avg±sd) | F-scan | R-scan | Statistics (avg±sd) |
| $J_{SC}$ (mA/cm$^2$) | 31.87 | 31.15 | 29.78 ±1.05 | 31.99 | 31.46 | 31.74 ±0.64 |
| $V_{OC}$ (V) | 0.784 | 0.785 | 0.80 ±0.033 | 0.858 | 0.860 | 0.840 ±0.008 |
| *FF* | 0.669 | 0.730 | 0.707 ±0.028 | 0.697 | 0.751 | 0.720 ±0.023 |
| *PCE* (%) | 16.74 | 17.85 | 16.96 ±0.77 | 19.14 | 20.32 | 19.17 ±0.56 |

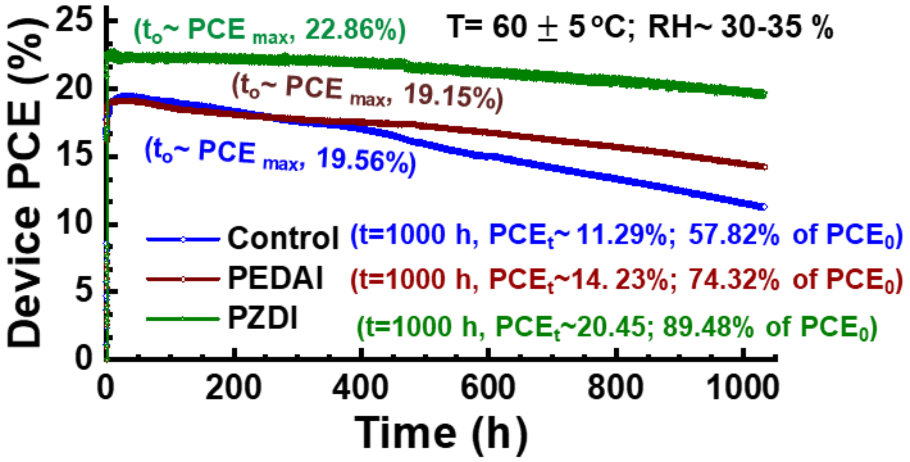


**Fig. S27.** Stability of the control, PEDAI, and PZDI passivated HPSCs. Non-normalized stability data operational tracking under MPPT conditions: T=60 ±5 °C; RH~ 30–35% (ISOS-L-2) (corresponding to Fig. 6a)

**Table S7.** Photovoltaic parameters of corresponding aged devices.

| Parameters/ Time (h) | control device | | | | PEDAI treated | | | | PZDI treated | | | |
|---|---|---|---|---|---|---|---|---|---|---|---|---|
| | $J_{SC}$ ($mAcm^{-2}$) | $V_{OC}$ (V) | *FF* | *PCE* | $J_{SC}$ ($mAcm^{-2}$) | $V_{OC}$ (V) | *FF* | *PCE* | $J_{SC}$ ($mAcm^{-2}$) | $V_{OC}$ (V) | *FF* | *PCE* (%) |
| 0 | 23.36 | 1.113 | 0.751 | 19.53 | 22.94 | 1.142 | 0.731 | 19.15 | 24.54 | 1.188 | 0.784 | 22.86 |
| 100 | 23.56 | 1.113 | 0.750 | 19.67 | 22.75 | 1.143 | 0.716 | 18.62 | 24.3 | 1.188 | 0.777 | 22.43 |
| 500 | 20.73 | 1.113 | 0.690 | 15.92 | 21.9 | 1.142 | 0.69 | 17.26 | 23.27 | 1.191 | 0.767 | 21.26 |
| 1000 | 15.36 | 1.114 | 0.660 | 11.29 | 18.55 | 1.145 | 0.67 | 14.23 | 22.66 | 1.194 | 0.756 | 20.45 |

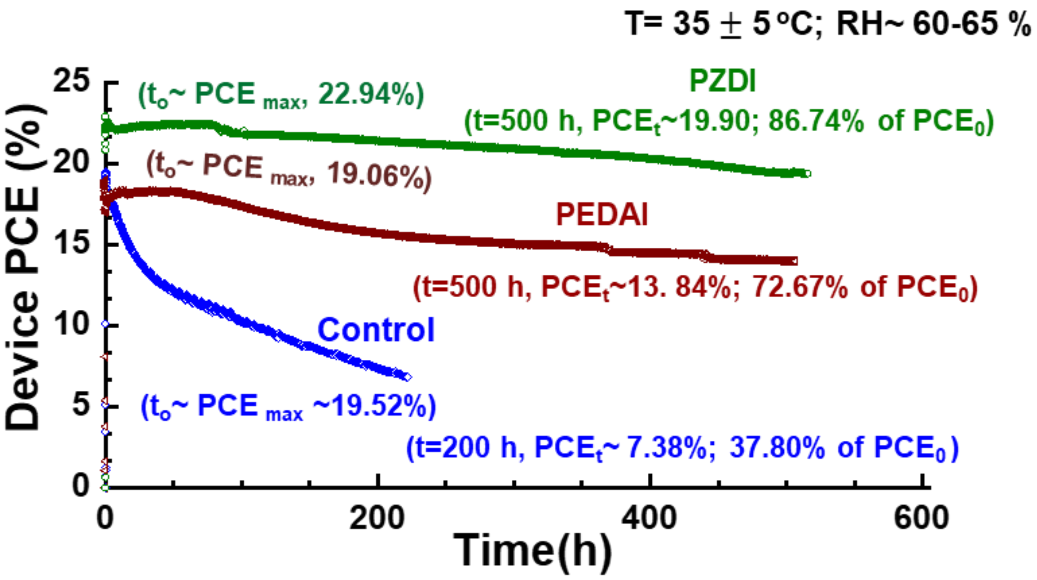


**Fig. S28.** Stability of the control, PEDAI, and PZDI passivated HPSCs. Non-normalized stability data operational tracking under MPPT conditions: T=35 ±5 °C; RH~ 60–65% (ISOS-L-3) (corresponding to **Fig. 6b**).

**Table S8.** Photovoltaic parameters of corresponding aged devices.

| Parameters/ Time (h) | Control device | | | | PEDAI treated | | | | PZDI treated | | | |
|---|---|---|---|---|---|---|---|---|---|---|---|---|
| | $J_{SC}$ ($mAcm^{-2}$) | $V_{OC}$ (V) | *FF* | *PCE* | $J_{SC}$ ($mAcm^{-2}$) | $V_{OC}$ (V) | *FF* | *PCE* | $J_{SC}$ ($mAcm^{-2}$) | $V_{OC}$ (V) | *FF* | *PCE (%)* |
| 0 | 23.62 | 1.104 | 0.717 | 19.52 | 22.98 | 1.146 | 0.724 | 19.07 | 24.5 | 1.188 | 0.788 | 22.94 |
| 100 | 19.16 | 1.03 | 0.52 | 10.23 | 21.28 | 1.145 | 0.712 | 17.35 | 23.74 | 1.188 | 0.775 | 21.86 |
| 200 | 16.16 | 1.016 | 0.45 | 7.38 | 19.94 | 1.143 | 0.69 | 15.73 | 23.35 | 1.189 | 0.772 | 21.43 |
| 500 | | | | | 17.76 | 1.144 | 0.68 | 13.82 | 21.81 | 1.191 | 0.766 | 19.90 |

**References**


1. Peng, J. *et al.* Nanoscale localized contacts for high fill factors in polymer-passivated perovskite solar cells. *Science* **371**, 390–395 (2021).

2. Peng, J. *et al.* Centimetre-scale perovskite solar cells with fill factors of more than 86 per cent. *Nature* **601**, 573–578 (2022).

3. Lin, X. *et al.* In situ growth of graphene on both sides of a Cu–Ni alloy electrode for perovskite solar cells with improved stability. *Nat Energy* **7**, 520–527 (2022).

4. Zhang, S. *et al.* Printable and Homogeneous NiO x Hole Transport Layers Prepared by a Polymer-Network Gel Method for Large-Area and Flexible Perovskite Solar Cells. *Adv Funct Mater* **31**, 2106495 (2021).

5. Peng, W. *et al.* Reducing nonradiative recombination in perovskite solar cells with a porous insulator contact. *Science* **379**, 683–690 (2023).

6. Bai, Y. *et al.* Initializing film homogeneity to retard phase segregation for stable perovskite solar cells. *Science* **378**, 747–754 (2022).

7. Tan, L. *et al.* Combined Vacuum Evaporation and Solution Process for High-Efficiency Large-Area Perovskite Solar Cells with Exceptional Reproducibility. *Advanced Materials* **35**, (2023).

8. Turren-Cruz, S.-H., Hagfeldt, A. & Saliba, M. Methylammonium-free, high-performance, and stable perovskite solar cells on a planar architecture. *Science* **362**, 449–453 (2018).

9. Li, Z. *et al.* 24.64%-Efficiency MA-Free Perovskite Solar Cell with Voc of 1.19 V Enabled by a Hinge-Type Fluorine-Rich Complex. *Adv Funct Mater* **33**, (2023).

10. Gharibzadeh, S. *et al.* Two birds with one stone: dual grain-boundary and interface passivation enables >22% efficient inverted methylammonium-free perovskite solar cells. *Energy Environ Sci* **14**, 5875–5893 (2021).

11. Pan, T. *et al.* Surface-Energy-Regulated Growth of α-Phase Cs0.03FA0.97PbI3 for Highly Efficient and Stable Inverted Perovskite Solar Cells. *Advanced Materials* 2208522 (2023).

12. Momma, K. & Izumi, F. VESTA 3 for three-dimensional visualization of crystal, volumetric and morphology data. *J Appl Crystallogr* **44**, 1272–1276 (2011).
