# Supplementary material for: Defect passivation in methylammonium/bromine free inverted perovskite solar cells using charge-modulated molecular bonding
Source: Nat Commun. 2024 Jan 29;15:882. doi: 10.1038/s41467-024-45228-9 (PMC10824754; doi:10.1038/s41467-024-45228-9)
Supplement: Supplementary file 3 — Description of Additional Supplementary Files [file 41467_2024_45228_MOESM3_ESM.pdf]

Description of Additional Supplementary Files

File name: Supplementary Data 1

Description: Single-crystal structure of (PZDI)2(PbI4)2.6DMSO (CCDC 2311444)

File name: Supplementary Data 2

Description: Single-crystal structure of (PZDI)3Pb2I7.6DMSO (CCDC 2311446)
