# Supplementary material for: Defect passivation in methylammonium/bromine free inverted perovskite solar cells using charge-modulated molecular bonding
Source: Nat Commun. 2024 Jan 29;15:882. doi: 10.1038/s41467-024-45228-9 (PMC10824754; doi:10.1038/s41467-024-45228-9)
Supplement: Supplementary file 6 — Solar Cells Reporting Summary [file 41467_2024_45228_MOESM6_ESM.pdf]

nature research

Corresponding author(s): Dhruba B. Khadka

Solar Cells Reporting Summary

Nature Research wishes to improve the reproducibility of the work that we publish. This form is intended for publication with all accepted papers reporting the characterization of photovoltaic devices and provides structure for consistency and transparency in reporting. Some list items might not apply to an individual manuscript, but all fields must be completed for clarity.

For further information on Nature Research policies, including our data availability policy, see Authors & Referees.

Experimental design

Please check: are the following details reported in the manuscript?

1. Dimensions

Item	Yes	No	Details
Area of the tested solar cells	☒	☐	1 cm2
Method used to determine the device area	☒	☐	Shadow mask

2. Current-voltage characterization

Item	Yes	No	Details
Current density-voltage (J-V) plots in both forward and backward direction	☒	☐	We provide current density-voltage (J-V) plots in both forward and reverse scans in Figure 2c and certified data Figure S10.
Voltage scan conditions *For instance: scan direction, speed, dwell times*	☒	☐	Scan speed: 0.05V/s ; in supporting information
Test environment *For instance: characterization temperature, in air or in glove box*	☒	☐	J-V test was done in air at room temperature (25-30 °C).
Protocol for preconditioning of the device before its characterization	☒	☐	We have placed our devices under UV light for glue curing for 30 min before J-V measurement.
Stability of the J-V characteristic *Verified with time evolution of the maximum power point or with the photocurrent at maximum power point; see ref. 7 for details.*	☒	☐	Maximum Power Point (MPP) Tracking for 120 seconds. Mentioned in experimental section.

3. Hysteresis or any other unusual behaviour

Item	Yes	No	Details
Description of the unusual behaviour observed during the characterization	☒	☐	No unusual behaviour and negligible hysteresis was observed.
Related experimental data	☒	☐	We provide Current density-voltage (J-V) plots in both forward and reverse scans in Figure 2c.

4. Efficiency

Item	Yes	No	Details
External quantum efficiency (EQE) or incident photons to current efficiency (IPCE)	☒	☐	We provide EQE spectra in Figure 2e.
A comparison between the integrated response under the standard reference spectrum and the response measure under the simulator	☒	☐	We compared JSC and current density integrated from EQE. The difference is below 3%. This is within the acceptable range. It is given in Figure 2e.
For tandem solar cells, the bias illumination and bias voltage used for each subcell	☐	☒	No tandem work is reported

5. Calibration

Item	Yes	No	Details
Light source and reference cell or sensor used for the characterization	☒	☐	Illumination under 1 sun with an AM1.5G spectral filter (100 mW/cm2) coupled with an MPPT system (Systemhouse Sunrise Corp.). The light intensity was calibrated by a silicon (Si) diode (BS-520BK). Mentioned in experimental section
Confirmation that the reference cell was calibrated and certified	☒	☐	Our solar simulator was calibrated by reference cell (BS-520BK) (calibrated and certified by BUNKOUKEIKI CO., LTD)

Calculation of spectral mismatch between the reference cell and the devices under test

☒ Yes ☐ No — On certified data

6. Mask/aperture

Size of the mask/aperture used during testing

☒ Yes ☐ No — For certification: 1.024

Variation of the measured short-circuit current density with the mask/aperture area

☒ Yes ☐ No — No significant variation was observed

7. Performance certification

Identity of the independent certification laboratory that confirmed the photovoltaic performance

☒ Yes ☐ No — Device certification was obtained from The National Institute of Advanced Industrial Science and Technology (AIST), Japan. It is registered as ISO / IEC 17025 accreditation laboratory (IAJapan ASNITE 0021 Calibration) according to international mutual recognition arrangements (MRA) for ILAC and APAC.

A copy of any certificate(s)
Provide in Supplementary Information

☒ Yes ☐ No — We provide certified data in Figure S11.

8. Statistics

Number of solar cells tested

☒ Yes ☐ No — We have fabricated more than 300 cells in 50 batches.

Statistical analysis of the device performance

☒ Yes ☐ No — Statistical analysis provided in Figure 2d and supporting information Figures S8, S9, and S10.

9. Long-term stability analysis

Type of analysis, bias conditions and environmental conditions
For instance: illumination type, temperature, atmosphere humidity, encapsulation method, preconditioning temperature

☒ Yes ☐ No — Experimental section: Operation stability were monitored under heat and moisture stress.
(a) 60 ±5 °C, relative humidity, 30–35% RH: ISOS-L-2
(b) 35±5 °C; 60–65% RH: ISOS-L-3
